# Nanofiber-expanded human umbilical cord blood–derived CD34^+^ cell therapy accelerates cutaneous wound closure in NOD/SCID mice

**DOI:** 10.1111/jcmm.12217

**Published:** 2014-01-23

**Authors:** Suman Kanji, Manjusri Das, Reeva Aggarwal, Jingwei Lu, Matthew Joseph, Vincent J Pompili, Hiranmoy Das

**Affiliations:** Stem Cell Research Laboratory, Cardiovascular Medicine, Davis Heart and Lung Research Institute, Wexner Medical Center at The Ohio State UniversityColumbus, OH, USA

**Keywords:** human umbilical cord blood, nanofiber-expanded CD34^+^ cells, cutaneous wound, NOD/SCID mice, collagen, MMPs

## Abstract

Nanofiber-expanded human umbilical cord blood–derived CD34^+^ cell therapy has been shown to have potential applications for peripheral and myocardial ischaemic diseases. However, the efficacies of expanded CD34^+^ cell therapy for treating cutaneous wounds and its mechanisms of action have yet to be established. Using an excisional wound model in non-obese diabetic/severe combined immune deficient mice, we show herein that CD34^+^ cells accelerate the wound-healing process by enhancing collagen synthesis, and increasing fibroblast cell migration within the wound bed. Concomitantly, reduced levels of matrix metalloproteinase (MMPs) such as MMP1, MMP3, MMP9 and MMP13 were detected in the wound beds of animals treated with CD34^+^ cells compared with vehicle-treated controls. CD34^+^ cells were found to mediate enhanced migration and proliferation of dermal fibroblast cells *in vitro*. Moreover, CD34^+^ cells secrete collagen in a serum-deprived environment. In mechanistic studies, co-culture of CD34^+^ cells with primary skin fibroblasts increased the expression of collagen1A1, a component of type 1 collagen, and decreased the expression of MMP1 in fibroblast cells in the presence of a proteasome inhibitor. Finally, CD34^+^ cell–mediated functions were transcriptionally regulated by the c-Jun N-terminal kinases pathway. Collectively, these data provide evidence of therapeutic efficacy and a novel mechanism of nanofiber-expanded CD34^+^ cell–mediated accelerated wound healing.

## Introduction

Wound healing is a complex biological process, requiring the involvement of various cell types and their mediators in an orchestrated manner, and characterized by an acute inflammatory phase followed by extracellular matrix (ECM) remodelling 1,2. Fibroblasts are the most important cells producing collagen-based ECM, which replaces the fibrin-based provisional matrix, and facilitate re-approximation of wound edges through their contractile properties as they migrate into the affected area 3. Thus, the processes of fibroblast migration, proliferation and ECM production within the wound bed are key steps in regeneration of functional dermis 4. As healing progresses, fibroblasts differentiate into myofibroblasts to promote wound contraction 3,4. In addition to co-ordinating processes, such as ECM synthesis, cell-to-cell interaction and cell-to-cytokine interactions, dermal fibroblasts not only repair wounds but also maintain the integrity of the skin 3,4. Any malfunction of the orchestrated cascades, such as impaired migration and proliferation of fibroblasts, will compromise the deposition of ECM and will result in delayed or impaired wound closure.

Matrix metalloproteinases (MMPs), such as MMP-1, -2 and -3, are the enzymes responsible for degradation and turnover of ECM, and spatio-temporal regulation of MMPs is critical for effective wound healing. Matrix metalloproteinases are also involved in tissue repair and remodelling processes such as inflammation, re-epithelialization and angiogenesis 5. An imbalance in activity of MMPs is often associated with chronically impaired wound healing 6. Production of MMPs is transcriptionally regulated, and requires activation from inactive precursors (proMMP) 7. Matrix metalloproteinase-1 cleaves type-I collagen by unwinding their triple-helix chains to make them susceptible to further degradation 8. In normal wound healing, MMP3 helps in epithelial cell migration, whereas MMP9 promotes inflammation and facilitates the migration of neutrophils, and MMP13 mediates endothelial cell migration. However, in the chronic wound, the levels of these MMPs are elevated 9,10. The activity of MMPs also depends on their interactions with ECM components and binding to endogenous inhibitors, such as tissue inhibitor of metalloproteinases (TIMP) 11.

Type-I collagen is an important ECM components of the skin required for normal growth, differentiation and wound repair 12. Collagen-I enhances ECM cross-linking, resulted in increased mechanical strength in the wound. Type-I collagen forms a triple-helix structure composed of two alpha 1 subunits and one alpha 2 subunit, encoded by the collagen 1A1 (COL1A1) and COL1A2 genes. Tightly regulated synthesis of these two moieties ensures a 2:1 ratio of COL1A1 and COL1A2 13.

Despite advances in wound care, cutaneous wound healing often demands significant long-term medical attention, and is responsible for huge expenses 14. Available medical interventions, such as systemic (*e.g*. hyperbaric oxygen therapy) or topical (*e.g*. growth factor; PDGF) therapy, and mechanical devices for wound protection, often fail to cure cutaneous wounds, leading to a significant number of peripheral amputations. Stem cells have long been recognized for their regenerative properties and viewed as potential therapeutics for healing wounds 15–17. The number of CD34^+^ stem cells obtained from a single cord is not sufficient for any preclinical or clinical application. Therefore, a variety of methods have been adopted, by which cord blood–derived stem cells can be expanded many fold without compromising their phenotype and stem cell characteristics. Previously, we have shown that the human umbilical cord blood–derived CD34^+^ cells can be expanded efficiently (almost 250-fold) on aminated nanofibers while preserving their stemness. In addition, after nanofiber expansion, CD34^+^ cells constitutively express high levels of a pro-migratory surface molecule (CXCR4), which helps them to mobilize to the challenged area. These umbilical cord blood–derived nanofiber-expanded CD34^+^ cells also show biological functionality in regenerating tissues in hind limb ischaemia and myocardial infarction models 18,19. However, both the efficacy of nanofiber-expanded CD34^+^ cells in cutaneous wound healing and their mechanisms of action have yet to be demonstrated.

In this study, using an excisional wound model in non-obese diabetic/severe combined immune deficient (NOD/SCID) mice, we show that CD34^+^ cells accelerate wound closure by enhancing collagen synthesis, and increasing fibroblast cell migration within the wound bed. CD34^+^ cell therapy–mediated accelerated wound closure was associated with reduced levels of MMPs. Accelerated wound closure might also be facilitated by enhanced ECM formation in the form of CD34^+^ cell–mediated secretion of collagen. Moreover, co-culture studies using primary dermal fibroblast cells indicate that the ability of CD34^+^ cells to enhance fibroblast migration, increase expression of COL1A1 and decrease expression of MMP1 in dermal fibroblasts is mediated through the c-Jun N-terminal kinases (JNK) pathway. Collectively, these data provide evidence for therapeutic efficacy and a novel mechanism of nanofiber-expanded CD34^+^ cell–mediated accelerated wound healing.

## Materials and methods

### CD133^+^ cell isolation

Human umbilical cord blood was freshly collected from The Wexner Medical Center at The Ohio State University after IRB approval, following written consent from donors, and was processed according to the protocol described earlier 18. Briefly, after gradient separation by the Ficoll centrifugation method, CD133^+^ cells were isolated using an AutoMACS device (Miltenyi Biotec, Auburn, CA, USA), and the purity of the isolated cells, as well as phenotypes after expansion, was determined by flowcytometry 18.

### *Ex-vivo* expansion of isolated cells

Freshly isolated CD133^+^ cells, which also co-express the CD34 molecule, were expanded by following the previously described protocol 18. Briefly, 800 CD133^+^ cells were cultured in a well of a 24-well plate coated with nanofiber mesh (a kind gift from Hai-Quan Mao, PhD, Johns Hopkins University, Baltimore, MD, USA) in 600 μl of StemSpan SFEM, a serum-free expansion medium (Stem Cell Technologies, Vancouver, BC, Canada) containing essential supplements. Cells were cultured at 37°C in an atmosphere containing 5% CO_2_ without changing culture medium, and harvested after 10 days. Before experiments, flow cytometry was performed to characterize the expanded cells. The majority of the expanded cells loses CD133 expression and retains CD34 expression.

### GFP labelling of CD34^+^ cells

Nanofiber-expanded cord blood–derived CD34^+^ cells were transfected with green fluorescence protein (GFP) containing vector (pmaxGFP) using the human CD34 cell specific Nucleofector kit (Amaxa Inc., Gaithersburg, MD, USA), following the manufacturer's protocol. After transfection, cells were cultured overnight in a serum-free complete medium and transplanted into the experimental mice.

### Fibroblast cell culture

A primary human dermal fibroblast cell line was established from skin punch biopsies of a healthy donor. Primary human dermal fibroblast cells (a generous gift from Dr. Heather M. Powell, Department of Materials Science and Engineering, Department of Biomedical Engineering, The Ohio State University, Columbus, OH, USA) were maintained in DMEM (Invitrogen Corporation, Carlsbad, CA, USA). DMEM medium was supplemented with 4% foetal calf serum (FCS; Sigma-Aldrich, St. Louis, MO, USA), 2 mM glutamine (Invitrogen Corporation), 5 μg/ml insulin (Sigma-Aldrich), 0.5 μg/ml hydrocortisone (Sigma-Aldrich), 0.1 mM ascorbic acid-2-phosphate (Sigma-Aldrich), 50 U/ml penicillin and 50 μg/ml streptomycin (Invitrogen Corporation), grown in 5% CO_2_ at 37°C, and were used within passages 3–6.

### Full-thickness excisional cutaneous wound model

All animal experiments were performed according to the protocols approved by the Institutional Animal Care and Use Committee of The Ohio State University, Columbus, OH. Six- to 8-week-old male NOD/SCID mice were used for this study and were purchased from Jackson Laboratory (Bar Harbor, ME, USA). Prior to generating a cutaneous wound, the mouse was anesthetized, the dorsum was clipped, hair was removed and the area was wiped with Betadine solution. A full-thickness wound was made on the dorsal skin in each mouse using 8-mm skin punch biopsy (Acuderm Inc., Fort Lauderdale, FL, USA).

### Transplantation of nanofiber-expanded GFP-labelled or unlabelled CD34^+^ cells

Ten-day nanofiber-expanded CD34^+^ cells (0.5 × 10^6^ cells/mouse) or GFP transfected (24 hrs prior to injection) CD34^+^ cells (0.5 × 10^6^ cells/mouse) in a 200-μl volume of serum-free DMEM media were injected into each mouse (*n* = 15), and media alone was injected as a control (*n* = 15) through lateral tail vein 2 hrs of post-wounding. Three mice were harvested from each group at days 3, 5 and 7. At each time-point, skin samples (wound and 2 mm of the surrounding skin) were harvested, and part of the sample was snap frozen in liquid nitrogen, while the other part was formalin fixed for further evaluations.

### Evaluation of wound area

Photographic images of wounds were taken on days 0, and 3, 5 and 7 after generation of wound using a digital camera (Sony cyber-shot DSC-H10, New York, NY, USA) from a fixed distance. Wound closure rate was measured by tracing the wound area onto a sheet of acetate paper on each day stated above following an earlier published method 20. The wound impressions were scanned and digitalized, and the areas were measured using the University of Texas Health Science Center at San Antonio image tool (Version 3.00) and converted to per cent wound. The percentage of wound closure was calculated as follows: (Area of wound on day 0 − Area of wound on day of examination)/Area of wound on day 0 × 100. The investigators measuring samples were blinded.

### Immunohistochemistry

Mice were killed at various time-points (days 3, 5 and 7) of the experiments, and skin tissues were harvested, so that part of the tissue was fixed in formalin-PBS buffer, paraffin-embedded and sectioned to generate wound-edge specimens of 4 μm diameter. After de-paraffinized, sections were stained with Masson's trichrome by standard procedures and examined under light microscopy. For immunofluorescence staining, antigen retrieval was performed with citrate buffer, pH to 6.0, and microwaving for 5 min., then cooling for 3 min. After non-specific blocking, specific staining was performed using alpha-smooth muscle actin (α-SMA; Sigma-Aldrich), Pro-collagen 1A1 (Santa Cruz Biotechnology, Inc., Dallas, TX, USA) Ab, or SM22α (Abcam, Cambridge, MA, USA) Ab, followed by incubation with secondary antibody alexa fluor 594–conjugated IgG or Texas red (Invitrogen, Molecular Probes, Carlsbad, CA, USA). Counterstaining was performed with DAPI (Invitrogen) and imaged using a fluorescent microscope (Nikon E800 with MetaMorph version 4.5 software, Universal Imaging Corp., Molecular Devices, LLC, Sunnyvale, CA, USA). The GFP staining procedure was carried out according to the protocol of VECTASTAIN Elite ABC kit (Vector Laboratories Inc., Burlingame, CA, USA) after using anti-GFP primary antibody (Zymed, Invitrogen). Immunohistochemical images were analysed using an image analysis software program (ImageJ, NIH, Bethesda, MD, USA).

### Fibroblast cell proliferation assay

The fibroblast cell proliferation assay was performed in a two-chambered 24-well plate. Human primary dermal fibroblasts (passage between 3 and 6) were seeded at 3 × 10^3^ cells/well in the lower chamber of a 24-well plate in DMEM complete media [10% fetal bovine serum (FBS)] and allowed to adhere, then starved overnight in DMEM (1% FBS) media. After serum starvation, 5 × 10^5^ CD34^+^ cells were added to the upper chamber in DMEM media containing 1% FBS and inserted into the fibroblast culture. Similar media without cells were inserted in a separate well as a control. After co-culture of the fibroblast and CD34^+^ cells for 48 hrs, the upper chambers were removed; fibroblast cells were trypsinized and counted with the trypan blue exclusion method using a Vi-Cell Cell Viability Analyzer (Beckman Coulter Inc., Brea, CA, USA). Fibroblast cell culture media were also frozen at −20°C for soluble collagen estimation later using a Sircol Collagen Assay kit.

### Scratch wound closure assay

The *in vitro* wound closure assay was performed in the lower chamber of a two-chambered 24-well plate using human dermal fibroblasts. Confluent human dermal fibroblasts were cultured in serum-deprived (1% FBS) DMEM for 24 hrs in the lower chamber of a 24-well plate, then wounded with a plastic micropipette tip having a large orifice. Scratched wells were washed with media to remove cell debris, and then either an empty control insert containing DMEM (1% FBS) media or CD34^+^ cells (5 × 10^5^ cells/well) DMEM (1% FBS) media containing insert were placed in the scratched fibroblast well. Photographs of scratched areas were taken at 0 and 48 hrs under a phase-contrast microscope. Wound closure was assessed by quantifying the number of fibroblasts migrated to the scratched region 21.

### Quantitative RT-PCR analysis

A quarter of a million fibroblast cells were seeded in a well of a 6-well plate, and serum-starved overnight. Then, the proteasome inhibitor, MG132 (10 μM), medium alone, CD34^+^ (0.25 × 10^6^) cells or CD34^+^ cells plus MG132 were then added to the fibroblasts and cultured for various time-points. MG132 was added 10 min. before addition of CD34^+^ cells. Total RNA was extracted from fibroblast cells after 6 and 12 hrs of culture using TRIzol reagent (Invitrogen) following the manufacturer's protocol. Real-time quantitative RT-PCR analysis was performed for MMP-1 and COL1A1 gene expressions. The reverse-transcription was performed with 1 μg of mRNA, and the ‘High Capacity cDNA Reverse Transcription Kit’ (Applied Biosystems, Foster City, CA, USA). One 20th of the cDNA was used for the real-time PCR analysis. Reactions were performed with SYBR Green PCR master mix (Applied Biosystems) in a Light Cycler 480 (Roche Applied Science, Indianapolis, IN, USA) detection system. The primers used were as follows: h-GAPDH, forward 5′-TTCGACAGTCAGCCGCATCTTCTT, reverse 5′-ACCAAATCCGTTGACTCCGACCTT; h-COL1A1, forward 5′-CAATGCTGCCCTTTCTGCTCCTTT, reverse 5′-CACTTGGGTGTTTGAGCATTGCCT; h-MMP1, forward 5′-ACAGAGATGAAGTCCGGTTT, reverse 5′-GAAGCCAAAGGAGCTGTAGAT. Expression levels of genes were normalized to GAPDH expression level.

### Western blot analysis

Western blot (WB) analysis for proteins isolated from wound tissue of animals with or without CD34^+^ therapy was performed by following the standard procedures. Primary antibodies used were for MMP1, α-SMA (from Santa Cruz, CA, USA), MMP3, MMP9, MMP13, SM22α (all from Abcam), β actin and GAPDH (both from Cell Signaling, Beverly, MA, USA). Mouse, rabbit IgG-HRP–conjugated (Cell Signaling) and goat IgG-HRP–conjugated (Santa Cruz, CA, USA) secondary Abs were used and specific bands were detected using enzyme-linked chemiluminescences (Pierce, Rockford, IL, USA). Densitometric analysis of developed bands was performed with UN-SCAN-IT (gel 6.1 version) software. Relative density was calculated using respective GAPDH/β-actin bands.

In a separate experiment, fibroblasts were cultured under serum-deprived (1% FBS) conditions. Total protein was extracted in lysis buffer containing protease and phosphatase inhibitors from five different conditions of fibroblast cultures, such as added MG132 (10 μM), CD34^+^ cells, CD34^+^ cells plus MG132 or MG132 plus SP 600125 (JNK Inhibitor II, 20 μM; from Calbiochem, Darmstadt, Germany), with medium alone serving as a control at both 6 and 12 hrs time-points. Twenty micrograms of total proteins was tested by WB analysis for levels of c-Jun and GAPDH (all from Cell Signaling) following the above-mentioned techniques.

### Total collagen assay

Fibroblasts were cultured in 1% FBS containing DMEM in the presence or absence of CD34^+^ cells. Cell culture supernatants were collected after 24 hrs of co-culture and stored at −20°C for later estimation of total collagen (types I–V). Total collagen was measured colorimetrically using a Sircol Collagen Assay kit (Bicolr Life Science Assays, Newtownabbey, UK) according to the manufacturer's protocol. Total collagen in the wound tissue (day 5 of post-wounding) was also measured colorimetrically using the similar kit and protocols. Briefly, Sircol dye reagent was added to the cell culture supernatants or tissue extracts, stirred for 30 min. at room temperature and centrifuged at 16,000 × *g* for 10 min. Absorbance of the bound dye was measured at 555-nm wavelength on a spectrophotometer. The amount of collagen protein in samples was adjusted to the total protein estimated by the BCA Protein Assay kit (Pierce). Collagen concentrations were expressed as microgram collagen per milligram of total protein.

### Statistical analysis

All values were expressed as mean ± SEM. Student's *t*-test was performed for comparison of data of unpaired samples. A *P* < 0.05 was considered significant.

## Results

### Isolation and expansion of human cord blood–derived stem cells

CD133^+^ cells were isolated from freshly collected human umbilical cord blood using the auto MACS system, and more than 95% cells were observed to be CD133^+^. The isolated CD133^+^ cells, which also co-express the CD34 molecule, were expanded on nanofiber matrices according to the earlier published protocols, and shown to retain stem cell phenotypes and characteristics 18. After 10 days of expansion on nanofiber-coated plates, the purity of the CD34^+^ cells was more than 90% as determined by flow cytometry 18.

### CD34^+^ cell therapy accelerate wound closure

**Figure 1:**
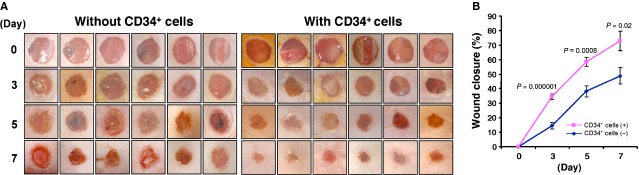
Nanofiber-expanded CD34^+^ cell therapy accelerates wound closure in non-obese diabetic/severe combined immune deficient (NOD/SCID) mice. (A) Morphological images of cutaneous wounds (8 mm punch biopsies) in NOD/SCID mice at day 0, 3, 5 and 7 of post-wounding with or without CD34^+^ cell therapy. (B) Graphical presentation of cumulative measurement of per cent wound closure on day 0, 3, 5 and 7 with or without CD34^+^ cell therapy. Results are shown as mean ± SEM (on day 3, *n* = 12; day 5, *n* = 9; day 7, *n* = 6).

The therapeutic potential of adult or foetal stem/progenitor cells for the treatment of cutaneous wounds has been noted in various preclinical models 15,17,22. To determine the therapeutic efficacy of nanofiber-expanded CD34^+^ cells in wound healing, we tested the ability of the nanofiber-expanded CD34^+^ cells to promote wound closure in an excisional cutaneous wound model generated in immunocompromised mice (NOD/SCID). Non-obese diabetic/severe combined immune deficient mice have often been used for transplantation of human cells as a result of lower graft rejection and are a well-established animal model for studying therapeutic efficacy for various disease states including cutaneous wounds 23. Using a cutaneous wound model, herein, we show that nanofiber-expanded CD34^+^ cell treatment significantly accelerates wound closure as early as day 3 of post-wounding and became more evident on day 7 of post-wounding in most of the animals studied by morphological image analysis (Fig. 1A). Cumulative analysis of wound closure data revealed that CD34^+^ cell therapy significantly enhanced the percentage wound closure in NOD/SCID mice compared with vehicle-treated control (Fig. 1B) at days 3, 5 and 7, indicating a potential use of these cells for cutaneous wound therapy.

### Nanofiber-expanded CD34^+^ cells recruited to the wound bed

**Figure 2:**
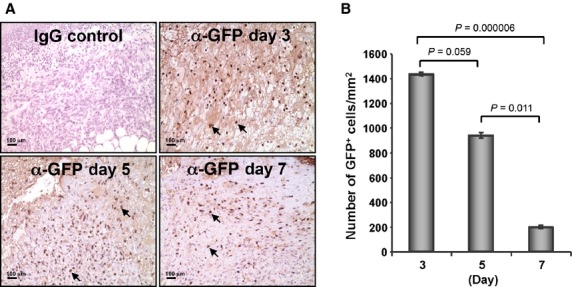
Nanofiber-expanded CD34^+^ cells were recruited to the wound bed. (A) Green fluorescence protein (GFP) was overexpressed on nanofiber-expanded CD34^+^ cells using Amaxa nucleoporation system, were assessed for homing to the wound bed at various time-points using α-GFP Ab by immunohistochemical methods. (B) Quantitative values of GFP^+^ cells were presented graphically. GFP^+^ cells were quantified by counting the cells in randomly chosen eight high-power microscopic fields within the wound-edge sections (*n* = 3) obtained from various time-points (day 3, 5 and 7). Data are presented as mean ± SEM.

To analyse the recruitment of CD34^+^ cells to the wound bed, nanofiber-expanded CD34^+^ cells were transiently transfected with pmaxGFP^+^ vector using the Amaxa electroporation system. More than 90% cells were GFP positive after 24 hrs and cell viability was more than 70%. GFP-positive CD34^+^ cells were transplanted within 24 hrs of transfection. Immunohistochemical analysis of wound tissue sections revealed that a substantial number of GFP^+^ cells were present throughout the healing process at days 3, 5 and 7 (Fig. 2A). Quantification of GFP^+^ cells showed that 1431 ± 13 GFP^+^ cells/mm^2^ were present at day 3, whereas the number of GFP^+^ cells decreased at day 5 and day 7 (936 ± 22 GFP^+^cells/mm^2^ at day 5; 196 ± 14 GFP^+^cells/mm^2^ at day 7) in the wound bed (Fig. 2B). These results indicate that nanofiber-expanded CD34^+^ cells were recruited to the wound bed after systemic administration and stayed within the wound bed during the wound-healing process.

### CD34^+^ cell therapy enhances fibroblast and myofibroblast levels in the wound bed

**Figure 3:**
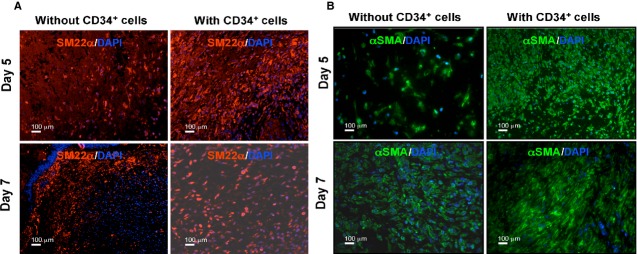
Enhanced fibroblast and myofibroblast cells in response to CD34^+^ cell therapy. Immunofluorescence-mediated detection of (A) fibroblast (SM22-α), and (B) myofibroblast (alpha-smooth muscle actin) cells in the wound bed of animals received CD34^+^ cells or without cells as a control at various time-points of therapy.

Fibroblast cells are one of the most important cell types in the skin, providing mechanical strength, and producing ECM, which in turn provides mechanical support for the cells within the dermis 3. Fibroblasts within the dermis differentiate into myofibroblasts, which are responsible for wound contraction. We wanted to investigate whether CD34^+^ cell therapy–mediated accelerated healing was because of the increased number of fibroblast/myofibroblasts present in the wound bed. As fibroblasts express SM22α and myofibroblasts express α-SMA, we have performed immunohistochemical analysis of the wound edges for those markers at various time-points, such as day 5 and 7 after CD34^+^ cells therapy or without cell therapy as a control. Immunohistochemical analysis revealed that expression of both α-SMA and SM22α markers, indicating fibroblasts and myofibroblasts, respectively, was increased in the wounds of animals treated with CD34^+^ cells compared with animals that did not receive any cell therapy at day 5 or 7 (Fig. 3A and B). Western blot analysis of total protein obtained from the wound tissues supported the conclusion that the mice treated with CD34^+^ cells express higher levels of SM22α compared with vehicle control mice (Fig. 5B). Collectively, these data indicate that higher abundance of fibroblasts and myofibroblasts was evident at the wound bed as a result of CD34^+^ cell therapy.

### CD34^+^ cell therapy increases collagen expression in the wound bed

**Figure 4:**
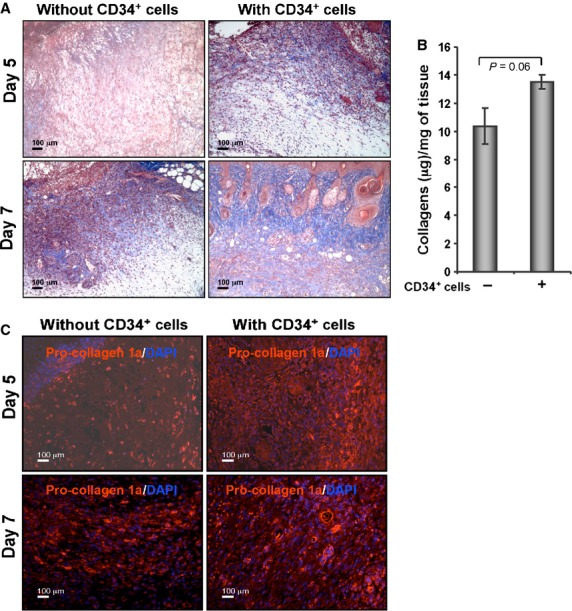
Enhanced collagen deposition in response to CD34^+^ cell therapy. (A) Masson's Trichrome staining (blue stain) was performed in the sections of wound edges at various time-points. (B) Graphical presentation of the amount of total collagen in wound skin lysates from the animals (*n* = 3) received CD34^+^ cells or media on day 5 after wounding, measured using a Sircol Collagen Assay kit. (C) Immunofluorescence detection of pro-collagen 1A1 (pro-COL1A1) in formalin-fixed paraffin sections of wound edges.

Collagen present within the ECM is responsible for the strength and resiliency of the skin, and mediates effective healing of the wounds 3,4. To investigate the amount of collagen present in the wound tissue, sections were subjected to Masson's trichrome staining. Masson's trichrome staining indicated that CD34^+^ cell therapy was associated with higher abundance of collagen at the wound bed compared with vehicle-treated control at day 5 or 7 (Fig. 4A). To quantify the total amount of collagens present in the tissues, homogenization of the total tissues was performed and supernatants were collected after centrifugation. Total collagen estimation assay (Sircol) was performed with tissue lysates, and it was found that collagen content was elevated in animals that received CD34^+^ cell therapy compared with animals that did not receive cells (Fig. 4B). However, the increased collagen content in the wounds of CD34^+^ cell-treated animals was not significantly different (*P* = 0.06) from control wounds. To study this effect further, immunohistochemical analysis of wound tissues was performed to investigate the presence of procollagen 1A1 (pro-COL1A1), the primary component of collagen 1. Immunohistochemical staining detected higher expression of pro-COL1A1 in the wound sections of animals that received CD34^+^ cell therapy compared with vehicle-treated control (Fig. 4C). This result indicates that CD34^+^ cell therapy mediates enhanced collagen deposition at the wound bed during the course of healing.

### CD34^+^ cell therapy reduces levels of matrix metalloproteinases

**Figure 5:**
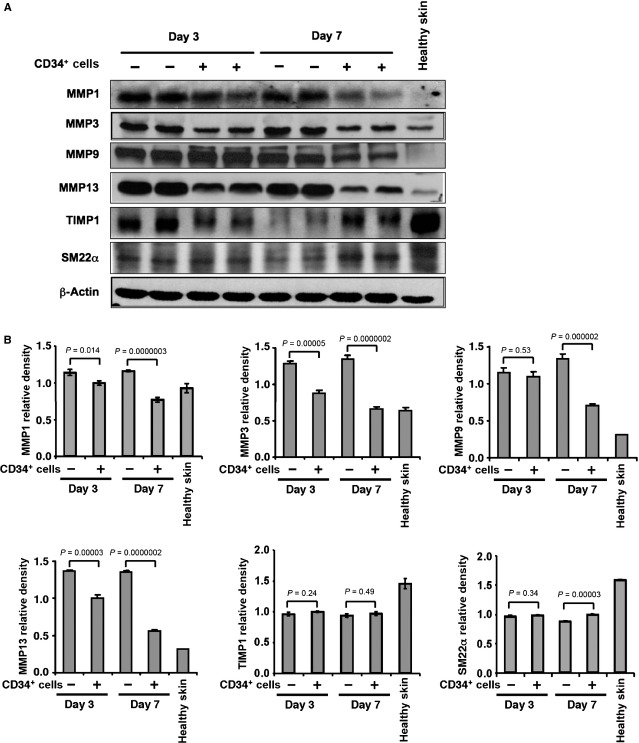
CD34^+^ cell therapy decreased matrix metalloproteinase (MMP) levels in wound tissues. (A) Representative western blot analysis of MMP-1, 3, 9, 13, tissue inhibitor of metalloproteinase 1, and SM22α protein levels in wound tissue lysates from mice received CD34^+^ cells or media as a control at day 3 and 7 after wounding. β-actin level was used as housekeeping protein for loading. (B) Quantitative values of each molecule were calculated by performing densitometric analysis of the targeted protein bands and graphically presented relative to β-actin amount. Results are shown as mean ± SEM (*n* = 3) within a representative of three independent experiments.

Matrix metalloproteinases play important role in many stages of wound-healing process, including inflammation, angiogenesis and remodelling 5, whereas TIMPs are the endogenous inhibitors of MMPs. Therefore, we sought to assess the expression levels of various MMPs and TIMP1 in the wound tissues of animals that had either received CD34^+^ cell therapy or vehicle only. Western blot analysis revealed a significant reduction (Fig. 5A and B) in the levels of MMP1, MMP 3, MMP 9 and MMP 13 in wound tissues of the animals treated with CD34^+^ cells at days 3 and 7 compared with the wounds of animals treated with vehicle only, except for the level of MMP 9 at day 3, where no significant difference was observed between the two groups of animals (Fig. 5B). However, CD34^+^ cell therapy did not increase TIMP1 levels significantly in the wound tissue compared with control wounds at any time-points tested. These results suggest that CD34^+^ cell therapy decreases MMP levels in the wounds, whereas TIMP1 is largely unaffected.

### CD34^+^ cells promote fibroblast cell migration and fibroblast cell number *in vitro*

**Figure 6:**
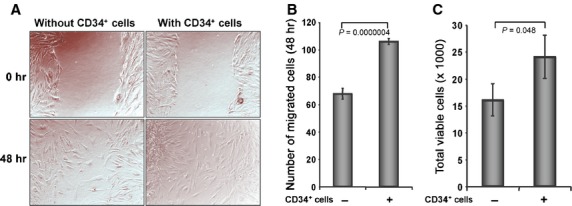
CD34^+^ cells enhance dermal fibroblast cell number and migration. (A) Cell migration assay was performed in two-chambered 24-well plate. Confluent dermal fibroblast was grown in lower chamber and monolayer was scratched with a p200 pipette tip. CD34^+^ cell containing inserts or without cell inserts (as a control) were placed on the top of well with scratched fibroblast cells. Images for each well were captured immediately after scratch and after 48 hrs of scratching. (B) Number of migrated fibroblast cells were counted in the presence or absence of CD34^+^ cells and presented graphically. (C) Number of fibroblast cells was counted from the bottom chamber of trans well (24-well) plate after 48 hrs of co-cultured with CD34^+^ cells or media. Non-adherent cells were removed by washing from the lower chamber before harvesting fibroblasts for analysis. Results are shown as mean ± SEM (*n* = 6) within a representative of three independent experiments.

To examine whether CD34^+^ cell therapy influences the biological effects of dermal fibroblasts, such as cell migration, we mimicked the cutaneous wound condition *in vitro* using a scratch wound to assess the effect of CD34^+^ cells on human dermal fibroblast cells. The scratch wound assay showed that the migration of dermal fibroblasts into the wound area was significantly higher (106 ± 8 cells) in the presence of CD34^+^ cells compared with the number of fibroblasts (68 ± 6 cells) in the absence of CD34^+^ cells (Fig. 6A and B). This result suggests that the presence of CD34^+^ cells largely influences the migration of fibroblast cells. The proliferation of fibroblasts is an important aspect of wound healing; therefore, we investigated the effects of CD34^+^ cells on the expansion of human fibroblast cells *in vitro*. The number of human dermal fibroblasts was significantly enhanced in the presence of CD34^+^ cells after 48 hrs of co-culture compared with the fibroblasts without CD34^+^cells (Fig. 6C), indicating that the presence of CD34^+^ cells may promote an increase in the numbers of human dermal fibroblasts.

### CD34^+^ cells synthesize/secrete collagen

**Figure 7:**
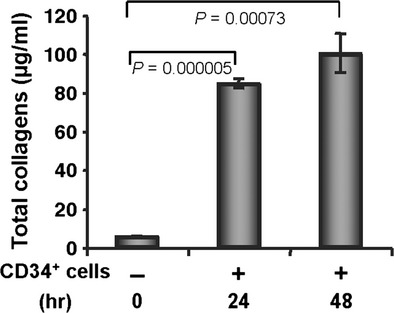
CD34^+^ cells secrete collagen. Total soluble collagen was measured using a Sircol Collagen Assay kit from the culture supernatants of CD34^+^ cells after 24 and 48 hrs of fasting, compared with baseline values, and graphically presented. Results are shown as mean ± SEM (*n* = 3) within a representative of three independent experiments.

To determine whether the CD34^+^ cells synthesize/secrete collagens to contribute to the accelerated wound-healing process, we tested total collagen synthesis/secretion in the cell culture supernatants at various time-points using a Sircol collagen assay kit without any added stimulus except serum deprivation. The total collagen assay revealed that CD34^+^ cells synthesize/secrete collagen under serum-deprived conditions, and that the amount of collagen is significantly higher at 24- or 48-hr time-points compared with the basal condition (0-hr time-point; Fig. 7). These results confirm that CD34^+^ cells not only mediate enhanced synthesis of collagen *in vivo* but also synthesize/secrete collagen by themselves to facilitate the wound-healing process.

### CD34^+^ cells influence expression of MMP1 and COL1A1 in dermal fibroblast cells

**Figure 8:**
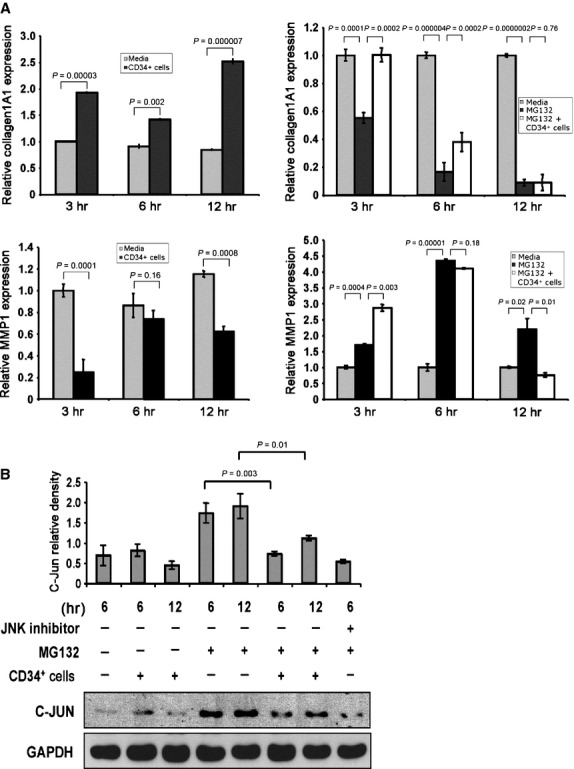
CD34^+^ cells enhance expression of collagen 1A1 and reduce expression of matrix metalloproteinase 1 (MMP1). (A) Graphical presentation of real-time PCR analysis data of collagen 1A1 (COL1A1, left, upper panel) and MMP1 (left, lower panel) gene expressions in cultured human primary dermal fibroblasts in the presence or absence of CD34^+^ cells at various timepoints. Similar experiments were performed for expression of COL1A1 (right, upper panel) and MMP1 (right, lower panel) genes in fibroblast cells in the presence or absence of proteasome inhibitor (MG132). Internal housekeeping gene β-actin was used as a reference for normalization. Data expressed as ±SEM (*n* = 3 in triplicate). The expression level of each target gene in fibroblast without any treatment was considered as base line. (B) Western blot analysis was performed for C-Jun in human primary dermal fibroblast cells cultured in the presence or absence of CD34^+^ cells (1:1 ratio and CD34^+^ cells were removed after culture) plus the presence or absence of proteasome inhibitor (MG132) and JNK inhibitor II (SP600125) at various time-points. GAPDH protein was evaluated as a housekeeping control. Quantitative values of C-Jun were calculated by performing densitometric analysis of the targeted protein bands and graphically presented relative to GAPDH amount. Results are shown as mean ± SEM (*n* = 3) within a representative of three independent experiments.

As dermal fibroblasts play a critical role in ECM formation 3, we investigated the mechanism by which CD34^+^ cells regulate the functionality of dermal fibroblast cells. Among the MMPs, MMP1 plays a predominant role in degrading dermal type-I collagen 8,24. Collagen 1A1 is the primary component of type-I collagen, a major component of ECM in the skin 24. A delicate balance between collagen synthesis and MMP activity is important for ECM turnover. Thus, we sought to further explore the effect of CD34^+^ cells on the expression of MMP1 and COL1A1 in dermal fibroblast cells using the quantitative RT-PCR method. Real-time RT-PCR analysis revealed that COL1A1 synthesis in dermal fibroblasts was significantly enhanced after co-culture with CD34^+^ cells at various time-points studied compared with the fibroblasts cultured alone (Fig. 8A, upper panel, left). On the other hand, MMP1 synthesis in dermal fibroblasts was significantly reduced after co-culture with CD34^+^ cells compared with the fibroblasts cultured alone. These results indicate that CD34^+^ cells influence the fibrogenic activity of dermal fibroblast cells (Fig. 8A, lower panel, left).

To confirm the specificity of CD34^+^ cells in regulating the expression of MMP1 and COL1A1 in dermal fibroblast cells, we assessed levels of MMP1 and COL1A1 in the presence of a proteasome inhibitor (MG132), a known inhibitor of COL1A1, and a stimulator of MMP1 expression, using RT-PCR methods 25. Quantitative RT-PCR analysis revealed that MG132 significantly reduced expression of COL1A1 in dermal fibroblasts, whereas COL1A1 expression was restored or partially restored by CD34^+^ cells at 3 or 6 hrs, respectively, under the same experimental conditions (Fig. 8A, upper panel, right). On the other hand, MG132 significantly increased the expression of MMP1 in dermal fibroblasts at all the time-points tested. On the other hand, CD34^+^ cells at 12 hrs significantly decreased MMP1 expression under similar experimental conditions (Fig. 8A, lower panel, right). This result indicates that CD34^+^ cells influence the expression of both MMP1 and COL1A1 in fibroblast cells.

### CD34^+^ cells suppress proteasome inhibitor–mediated c-Jun levels in dermal fibroblasts

To specifically define the molecular pathway involved in this process, we chose to examine the JNK pathway, as it is known that MG132 specifically induces an increase in c-Jun level, which primarily regulates decreased COL1A1 and increased MMP1 synthesis 25. Therefore, we investigated whether CD34^+^ cells have any effect on levels of c-Jun molecule in dermal fibroblasts in the presence or absence of MG132. Western blot analysis revealed that c-Jun expression in fibroblast cells was increased in the presence of MG132, whereas in the presence of both CD34^+^ cells and MG132, the level of c-Jun was decreased significantly at various time-points tested (Fig. 8B). A JNK inhibitor also reduced the level of c-Jun in the presence of MG132, confirming the involvement of the JNK pathway (Fig. 8B).

## Discussion

Although significant advancement has been made in all aspects of wound-healing therapy, still a large number of amputations occur each year as a result of the refractory wounds, which impose a burden on the economy and patient's lives 26. Cell-based therapies show promise in healing wounds in various models; however, mechanistic pathways have not yet been investigated in detail other than angiogenesis 15,22. A limitation in procurement of an adequate number of stem cells has long hindered the success of cell-based therapeutic intervention for degenerative diseases. However, our established CD34^+^ cell expansion method provides an adequate number of stem cells for preclinical evaluations in various ischaemic and degenerative disease models 18,19,27. However, their potential for treating wounds and their underlying mechanisms of action are previously unexplored. Recently identified telocytes, resemble with ‘dendritic cells’, which are also positive for CD34 marker, present in the human dermis, might play role in skin regeneration 28. However, morphologically, telocytes are significantly different from nanofiber-expanded cord blood–derived CD34^+^ cells, which remain round in shape after nanofiber expansion. The human umbilical cord blood–derived stem cell, as a unit, has been the basis for an important regimen for treating multiple haematological disorders for more than five decades 29,30. In addition, human cord blood–derived stem cells are currently being used for regenerative studies as they possess minimal oncogenic transformation capabilities, and display stable telomeres, which also serve to protect against potential oncogenic transformation 31–33. Moreover, we have not observed oncogenic transformation of transplanted CD34^+^ cells in various preclinical models in our previous studies 18,19,27,34. Using peripheral blood-derived CD34^+^ cells in a wound-healing model, another laboratory also did not show any oncogenic transformation of CD34^+^ cells 22, suggesting that CD34^+^ cell transplantation is safe.

In our current study, nanofiber-expanded human umbilical cord blood–derived CD34^+^ cell therapy improved wound healing and accelerated cutaneous wound closure in NOD/SCID mice (Fig. 1A and B), consistent with the previous observations of blood-derived stem cell–mediated accelerated wound healing 15,22. This accelerated wound closure was mediated by efficient recruitment of nanofiber-expanded CD34^+^ cells to the wound bed, as a substantial number of GFP^+^ CD34^+^ cells were observed at the wound bed after tail vein administration (Fig. 2A and B). The recruitment of CD34^+^ cells to the wound bed was facilitated by the constitutive expression of CXCR4 on the surface of CD34^+^ cells after nanofiber expansion 18, which correlated well with previous observations where expression of CXCR4 on the surface of haematopoietic stem cells helps their preferential migration to the inflammatory or ischaemic areas 35,36.

An open wound injury requires the well-orchestrated integration of complex biological and molecular events for its healing 1,2. It is well known that both fibroblasts and myofibroblasts play critical roles in the wound-healing process. Specifically, the traction forces of fibroblasts and coordinated contraction of myofibroblasts facilitate accelerated wound contraction and closure 37. In our present study, we observed that animals, treated with CD34^+^ cells, in comparison with control, have higher abundance of myofibroblasts, expressing α-SMA 12, at the wound bed (Fig. 3). This observation correlated well with accelerated wound closure in CD34^+^ cell–treated animals (Fig. 1). Myofibroblasts in cutaneous wounds are generally believed to evolve from resident fibroblasts in the dermis and subcutaneous tissues surrounding the wound 38. Along with other cells, fibroblast cells express the SM22α protein 39. We have found a higher abundance of SM22α-positive cells at the wound bed of CD34^+^ cell–treated animals (Figs. 3 and 5B), indicating a higher number of fibroblast cells at the dermis area of wounds, which probably, later on, differentiate into myofibroblasts, as indicated by higher expression of α-SMA–positive cells. However, in pathological conditions such as diabetes, fibroblasts fail to respond to injury as a result of impaired proliferation capabilities 40. Hence, improving functionality in fibroblast migration and proliferation could be a strategy to achieve normal wound healing. In this study, we have further demonstrated *in vitro* that CD34^+^ cells could enhance migration and most likely proliferation of dermal fibroblasts at the wound area (Fig. 6 A–C). However, this will be tested more conclusively in future studies where the use of proliferation markers will clearly demonstrate the capability of CD34^+^ cells to mediate fibroblast proliferation.

Fibroblasts and myofibroblasts also play pivotal roles in the continuous deposition of ECM proteins, such as collagen types I–VI, which are essential components for wound healing 12,41. Interestingly, we found a higher intensity of collagen staining in the wounds of animals treated with CD34^+^ cells compared with those without CD34^+^ cells (Fig. 4A), indicating a role of CD34^+^ cells in promoting collagen formation. After quantifying, the total amount of collagen present in the wound tissues, we found that the collagen content was much higher in the wounds of animals treated with CD34^+^ cells compared with the controls, but not statistically significant (Fig. 4B). To identify the sources of collagens, we conducted a test *in vitro* and found that CD34^+^ cells themselves secrete a significant amount of collagen (Fig. 7). Type-I collagen is a major component of ECM in the skin. We also observed a higher abundance of procollagen 1a in the wounds of animals treated with CD34^+^ cells compared with the controls (Fig. 4C). This *in vivo* observation was confirmed *in vitro* when human fibroblasts were co-cultured with CD34^+^ cells, after which COL1A1 expression was significantly increased compared with fibroblasts alone (Fig. 8A). In addition, in the presence of the specific proteasome inhibitor MG132, the expression of COL1A1 was decreased, which was consistent with earlier studies 25. However, the expression of COL1A1 by dermal fibroblasts in the presence of MG132 was recovered with the co-culture of CD34^+^ cells (Fig. 8A), indicating a positive regulation of collagen synthesis and ECM formation by CD34^+^ cells during the wound-healing process.

Matrix metalloproteinases are primarily involved in the remodelling of ECM. However, uncontrolled activity of MMPs is often associated with delayed, deficient or impaired wound closure 42. Reports show that the expression levels of MMPs are markedly elevated in chronic ulcers compared with normal wounds 6,43,44, as these proteinases degrade essential ECM components, integrin receptors, growth factors and their receptors, which are essential components for healing 45. In our present study, CD34^+^ cell therapy resulted in decreased levels of MMP-1, -3, -9 and -13 in wounds (Fig. 5), and was highly associated with faster wound healing, due at least in part to the limited degradation of critical ECM components and other essential factors. Activity of these MMPs is known to depend on binding to endogenous inhibitors such as TIMP 11, and higher TIMP expression, thus, may contribute to higher ECM turnover. We observed higher expression of TIMP1 in some CD34^+^ cell–treated wound tissues, but did not find a significant difference from control wounds (Fig. 5A and B). Hence, the impact of CD34^+^ cell therapy on the regulation of TIMP1 is inconclusive. Furthermore, MMP-1, in particular, has a distinct role in cleaving the triple helix of type-I collagen, allowing the chains to unwind and become susceptible to further degradation 8. Not only does CD34^+^ cell therapy have the ability to down-regulate MMP1 protein at the wound tissue (Fig. 5A and B) but these CD34^+^ stem cells also have the ability to suppress the expression of MMP1 in dermal fibroblasts *in vitro* (Fig. 8A), even in the presence of a proteasome inhibitor (Fig. 8A), known to increase the synthesis of MMP1 in dermal fibroblasts 25.

In summary, CD34^+^ cell therapy accelerates wound healing by enhancing collagen synthesis and reducing MMP production in the wound bed *via* migration of fibroblasts into the wound bed. Although the potential clinical application of CD34^+^ cells remains to be investigated, this study serves as a proof of concept that CD34^+^ cell therapy could be considered a useful strategy for the treatment of refractory wounds where wound contraction, ECM deposition and tissue remodelling are severely compromised.
